# Arthroscopic Midcarpal Tendon Interposition: A New Technique for Capitolunate Constraints

**DOI:** 10.1016/j.eats.2021.12.020

**Published:** 2022-04-22

**Authors:** Jean-Baptiste de Villeneuve Bargemon, Clément Prenaud, Christophe Mathoulin, Lorenzo Merlini

**Affiliations:** a Hand Surgery and Limb Reconstructive Surgery Department, La Timone Adultes Hospital, Aix Marseille University, Marseille, France; b Department of Orthopaedic Surgery, Public Assistance Hospital of Paris, Bobigny, France; c International Wrist Center, Clinique Bizet, Paris, France

**Keywords:** arthroscopy, interposition, SLAC, SNAC, tendon, wrist

## Abstract

Conflicts of the capitolunate, causing midcarpal pain from friction can be isolated (e.g., avascular necrosis of the proximal pole of the capitate [AVNC], palmar midcarpal instability [PMCI]) or form part of a framework of more complex osteoarthritis phenomena (e.g., scaphoid pseudarthrosis [SNAC], and lesions of the scapholunate capsuloligamentous complex [SLAC]). We group in the term “conflict” all of the causes (with intact cartilage or not) causing midcarpal pain by friction. Treatment by capitolunar arthrodesis can be effective, but inevitably stiffening. In other more specific cases (i.e., AVNC), replacement of the proximal pole of the capitate with a synthetic implant or a tendon has shown variable results. In this work, we propose a management of these conflicts with a conservative arthroscopic technique, including capitolunate tendon interposition. We describe arthroscopic midcarpal tendon interposition (AMTI) for capitolunate conflicts. This technique prevents stiffness due to arthrodesis, but good experience in wrist arthroscopy is required to perform this operation.

## Introduction

**Fig 1:**
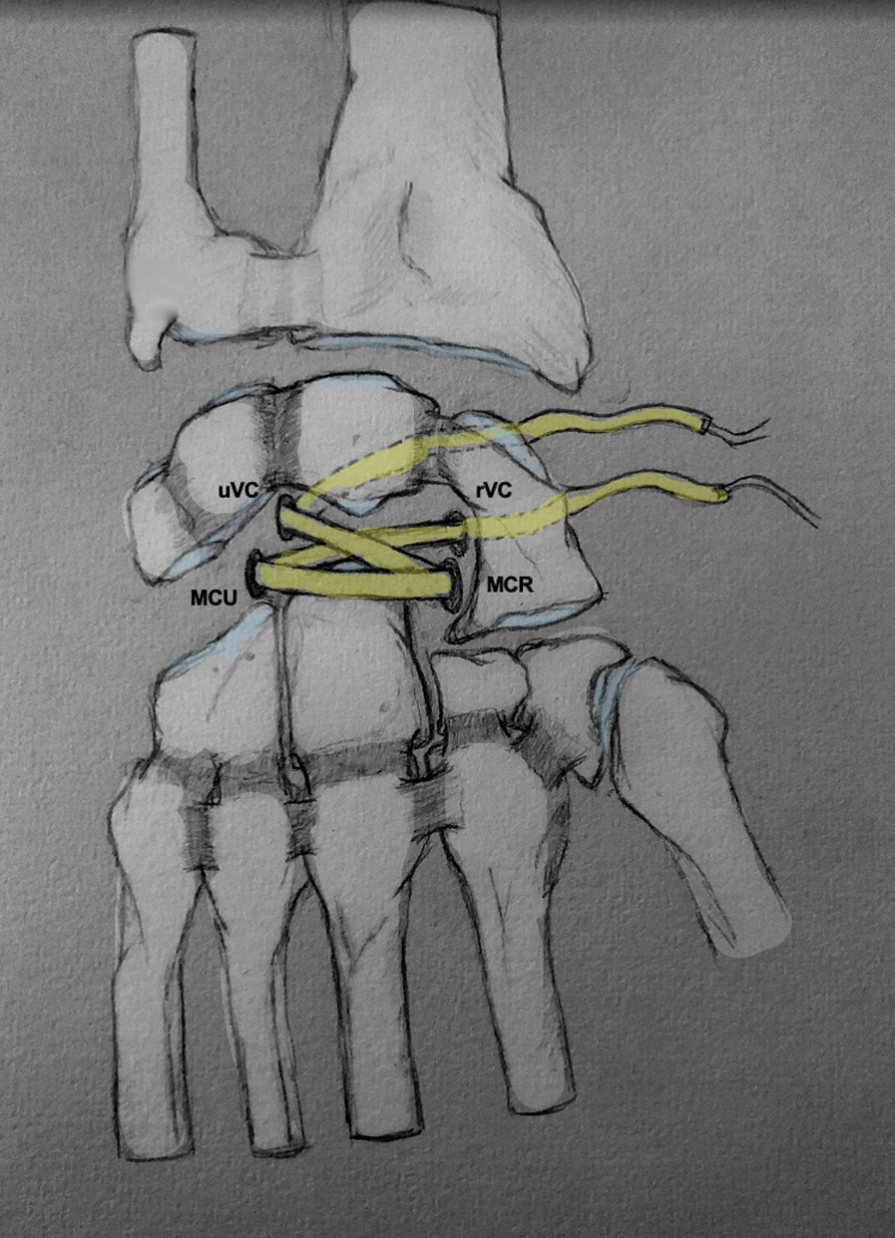
Schematic representation of arthroscopic midcarpal tendinous interposition.

The management of capitolunate conflicts presently remains controversial, although several solutions are possible. Capitolunate arthrodesis (CLA) appears to be an effective method for the management of pain and strength, at the cost of loss of range of motion.^1^ This solution offers good results in osteoarthritic capitolunate lesions in the context of more general osteoarthritis phenomena, such as carpal collapse due to scaphoid pseudarthrosis (SNAC) or lesions of the scapholunate capsuloligamentous complex (SLAC). This procedure can be performed arthroscopically and has shown better functional results than a four-corner arthrodesis^2^ in terms of pain, strength, and range of motion. However, during conflict pain without arthritic lesions, the functional consequences of this arthrodesis procedure remain significant. With necrosis of the proximal pole of the capitate, pain may be present in the absence of osteoarthritic lesions. Although this is a rare pathology (the largest cohort reported is 6 patients^3^), several treatments have been proposed, such as scaphocapitolunate or four-corner arthrodesis,^3^ replacement of the proximal pole with a pyrocarbon implant,^4^ or an anchovy (tendon tied on itself )^5^ tendon with fairly variable results, and sometimes with few backup solutions in the event of failure. The aim of this article is to present a conservative treatment option for capitolunate conflicts, illustrating an arthroscopic technique of tendon capitolunate interposition (Fig 1).

## Surgical Technique (Video 1)

### Patient Preparation

The procedure is performed on an outpatient care basis under regional anesthesia using a tourniquet. The patient’s arm is secured to the arm board and finger traps are used to apply 5–7 kg (11–15.5 lbs) traction along the arm’s axis.

### Graft Harvesting

First, harvesting of the ipsilateral PL is performed using a stripper (Tendon Stripper, Arthrex Fort Myers, FL). The graft is harvested through a small incision at the distal flexion crease of the wrist joint at the base of the carpal tunnel. The PL is then prepared with sutures on each free end (Vicryl 3.0).

### Arthroscopic Exploration

The scope (30°, 2.4-mm diameter; Karl Storz, Tutlingen, Germany) is introduced in the 3-4 portal and the shaver (Karl Storz; 2.9) in 6R portal. The first phase of the arthroscopic procedure consists of complete synovectomy with a shaver, reversing the shaver and scope positions. The scope is then introduced through the midcarpal ulnar (UMC) portal, and the shaver is introduced through the midcarpal radial (RMC) portal to debride the joint. The shaver and scope are reversed to debride the remainder of the joint. We confirmed the presence of avascular necrosis of the capitate in its proximal pole.

### Volar Central Portal

We used a palmar midcarpal portal to perform this interposition, as described by Corella et al.^6^ This portal is centrally located at the anterior horn of the lunate between the median nerve, pollicis longus (PL), flexor pollicis longus (FPL), the flexor carpi radialis (FCR) radially, and the finger flexors ulnarly. Then, two capsular perforations are performed: one radial volar central (rVC) and the other ulnar volar central (uVC).

### Midcarpal Interposition

**Fig 2:**
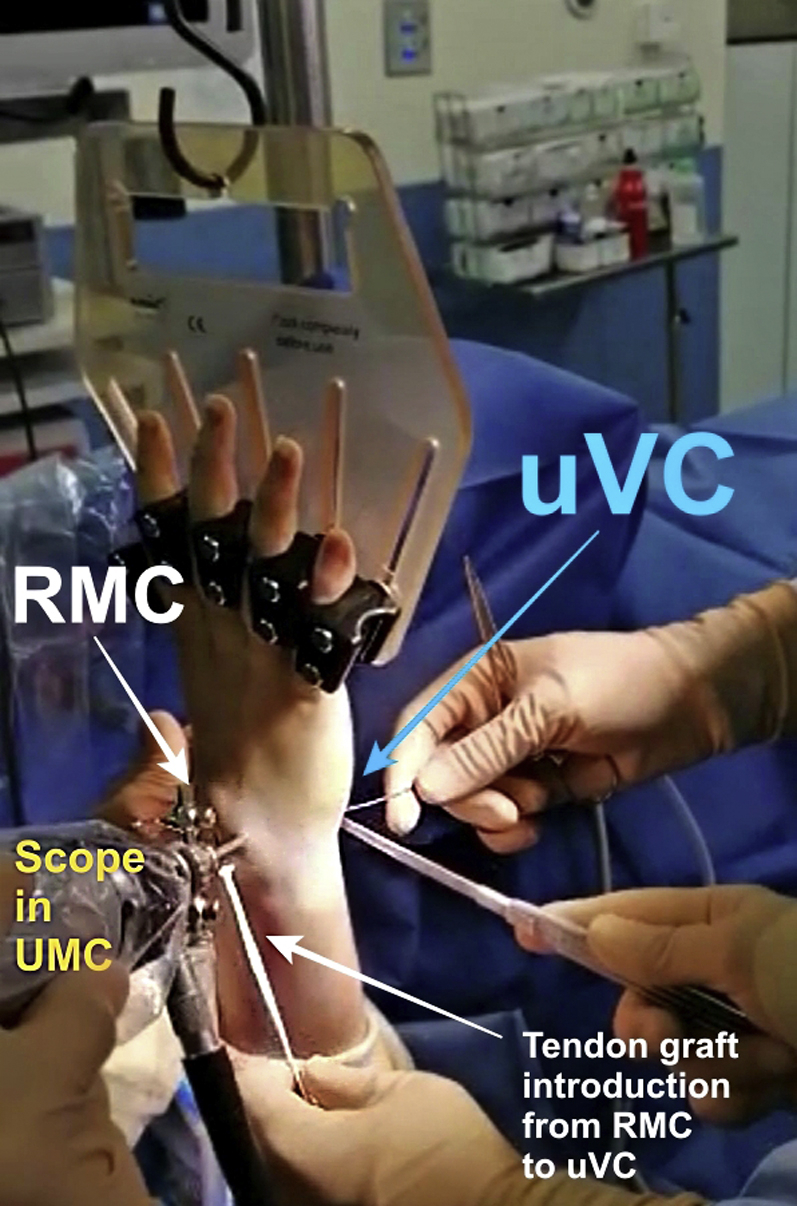
Graft introduction from RMC to uVC (extra-articular view). Arthroscopic midcarpal tendinous interposition (AMTI) was performed on a right wrist. Patient was in the supine position. The tourniquet was inflated to 250 mmHg. The traction was applied by a Finochietto hand or by Chinese fingers. It should be noted that the tendon can be introduced from RMC to uVC, as well as from uVC to RMC. RMC, radial midcarpal portal; uVC, ulnar volar central portal.

The graft is introduced from the uVC portal to the RMC portal using mosquito forceps. The tendon can be introduced from uVC to RMC or in the opposite way for the first step without it being important for the remainder of the surgery (Fig 2).

**Fig 3:**
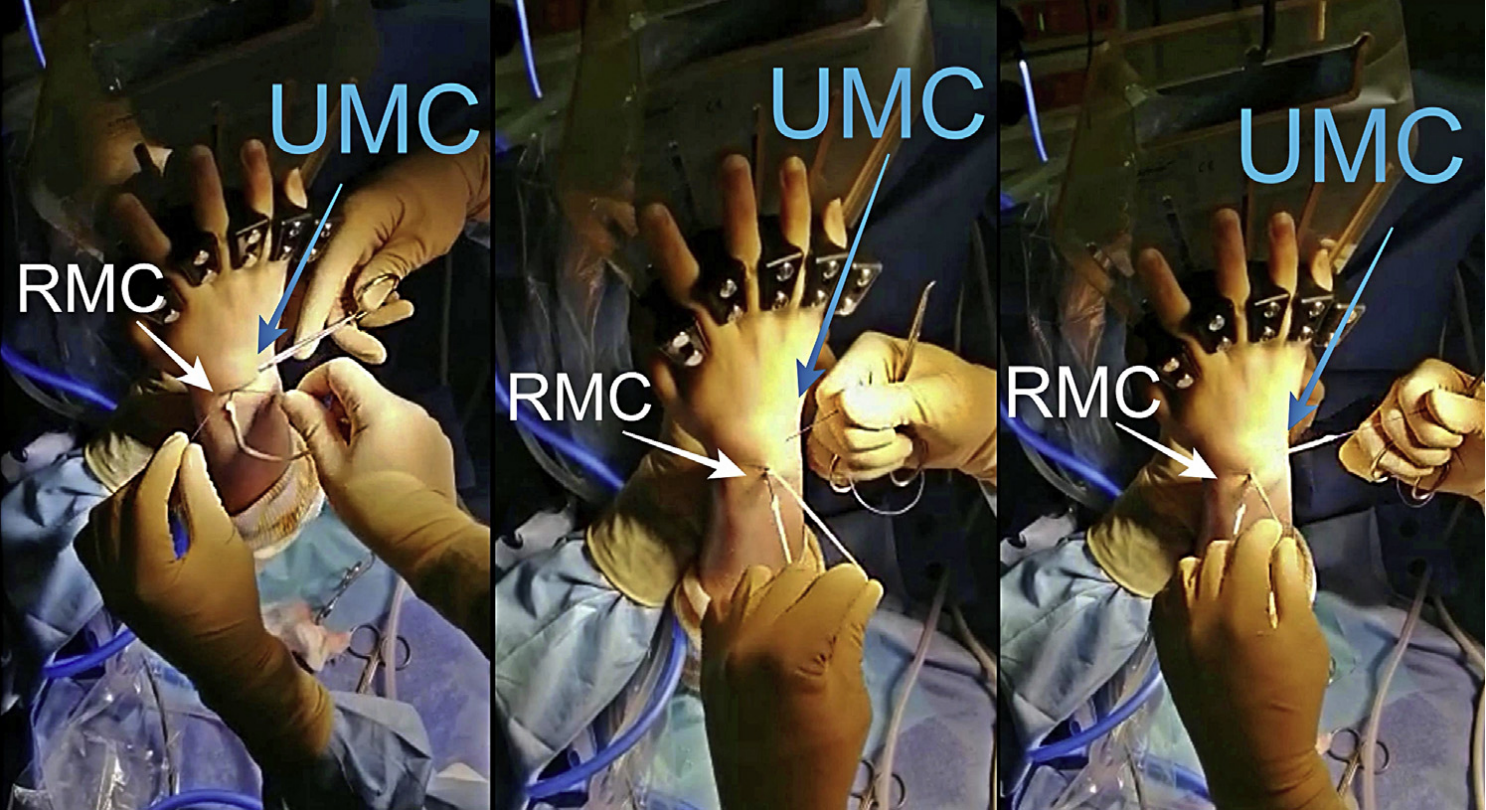
Graft passage from RMC to UMC in extra-articular way (extra-articular view). A first empty passage with mosquito forceps allows the creation of a space for dissection between the capsule and extensors in order to avoid any risk of tendon being caught in the interposition. RMC, radial midcarpal portal; UMC ulnar midcarpal portal.

**Fig 4:**
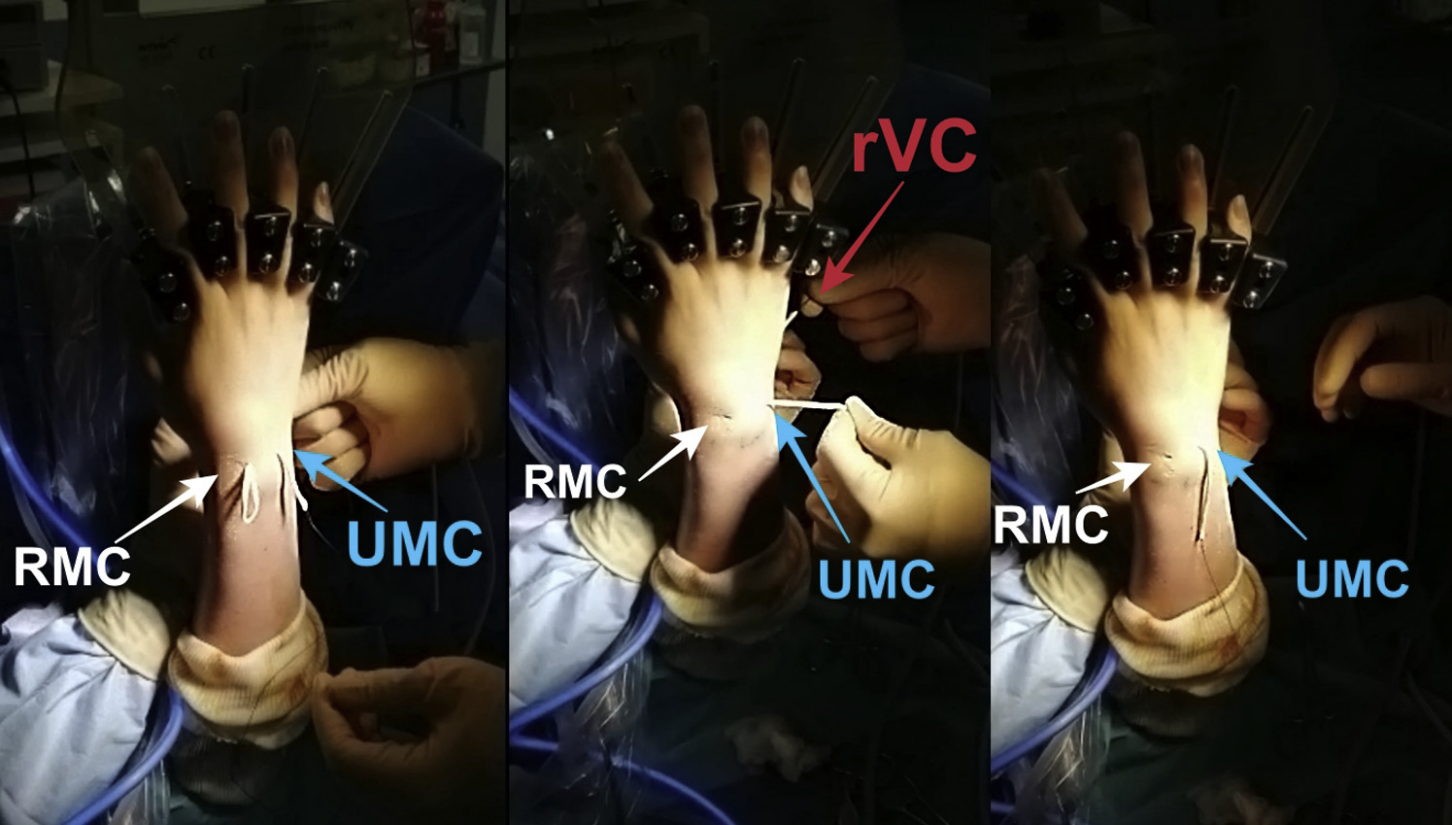
The graft is stretched by both ends (uVC and UMC) (extra-articular view). The tendon graft should no longer appear at the RMC portal. UMC, ulnar midcarpal portal; uVC, ulnar volar central portal.

**Fig 5:**
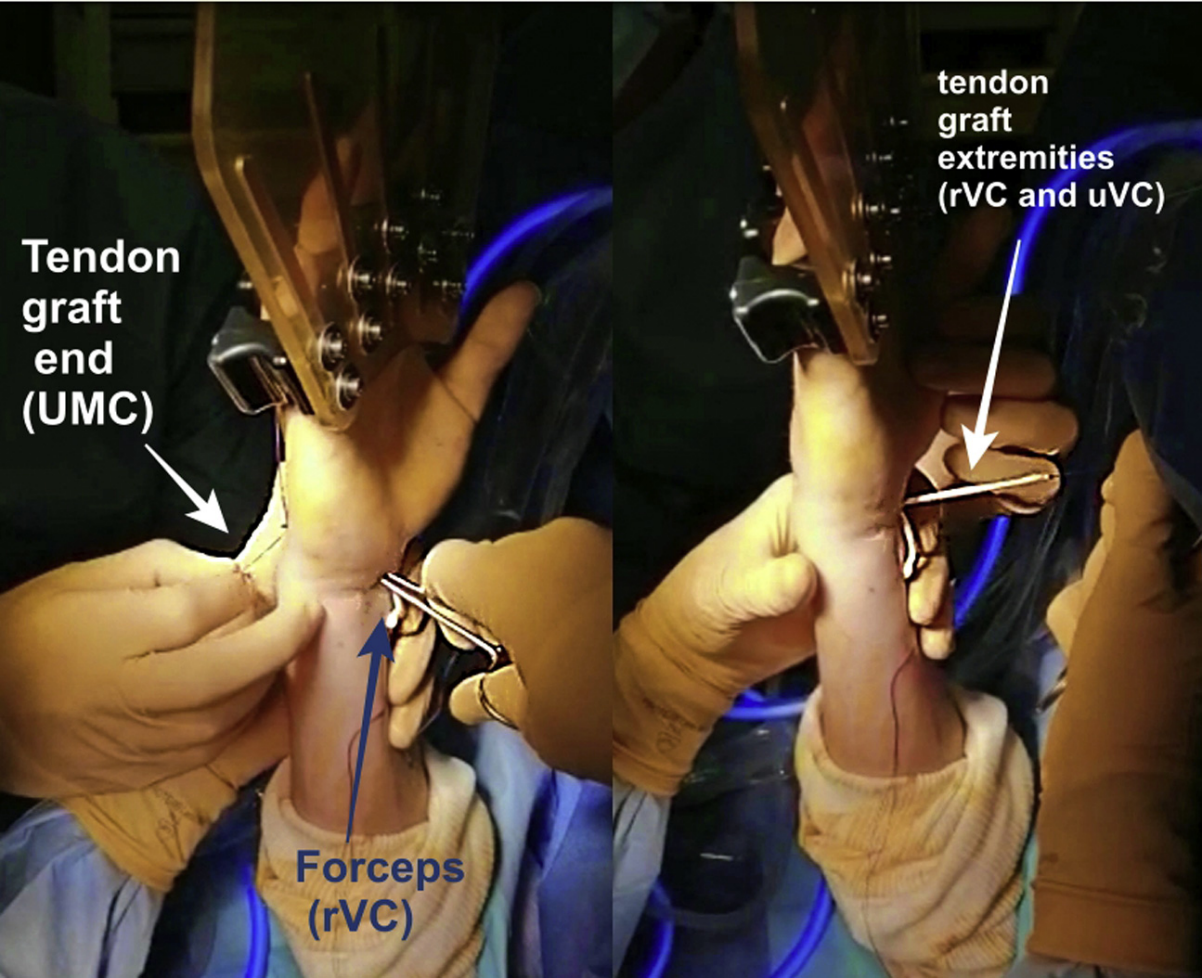
Graft passage from UMC to rVC in intra-articular way (extra-articular view). The two ends of the tendon graft are, therefore, palmar after having crossed at the level of the capitolunar joint. rVC, radial volar central portal; UMC, ulnar midcarpal portal.

**Fig 6:**
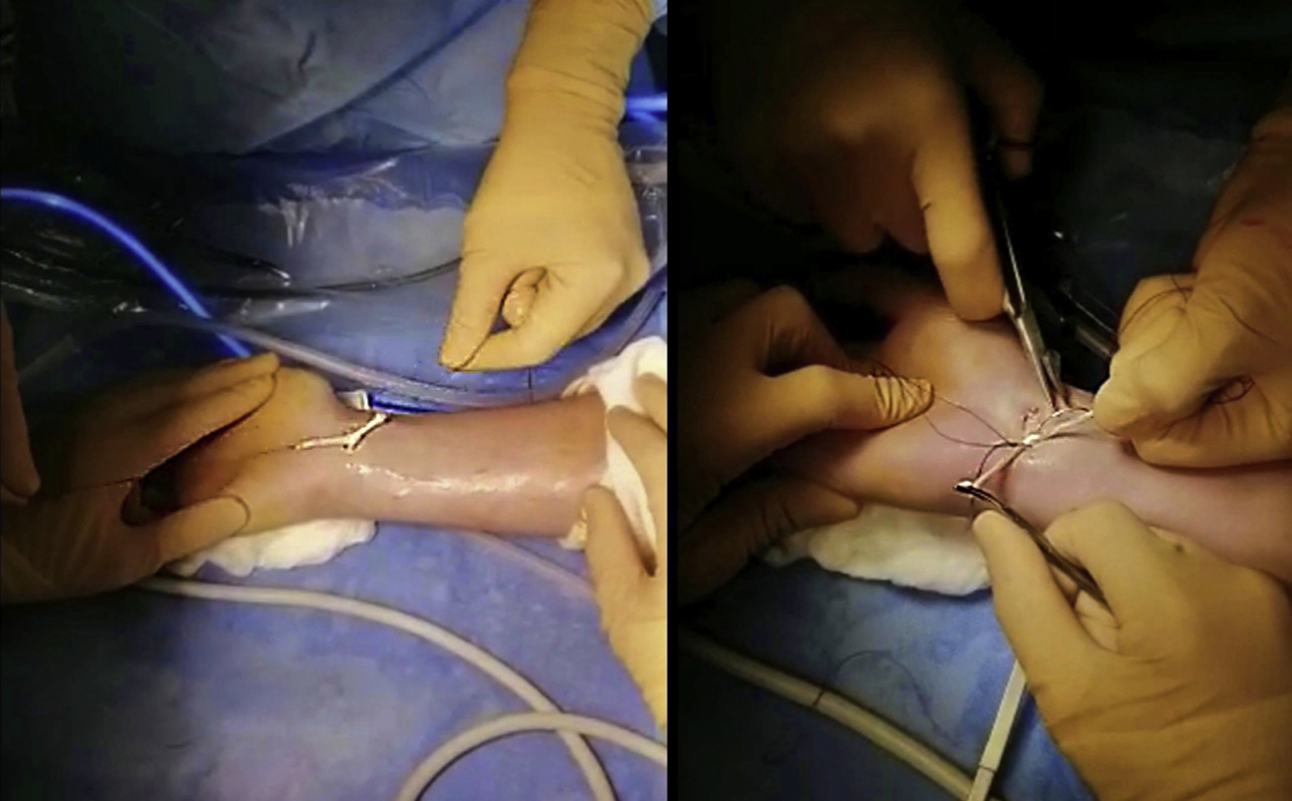
Final suture (extra-articular view). The two tendon strands are tied with each other. The tendon node will be tilted intra-articularly using a forceps.

**Fig 7:**
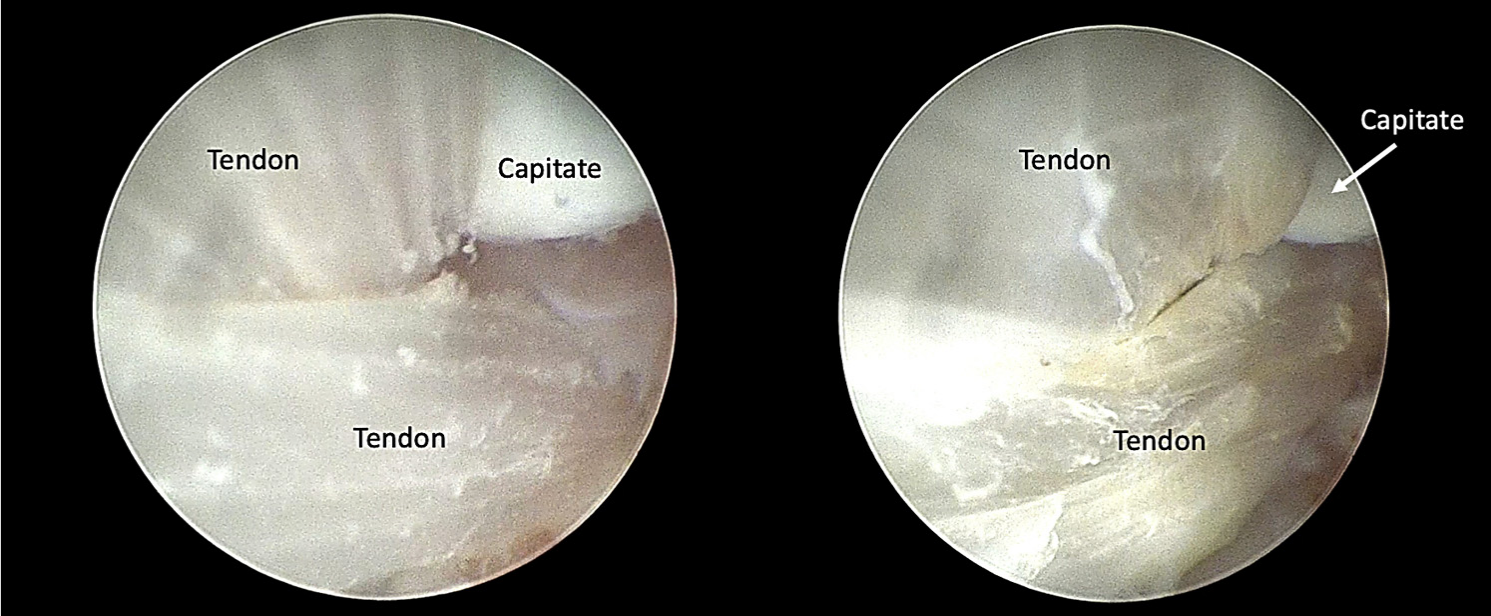
Scope in UMC, probe in RMC (intra-articular view). The correct position of the interposition prevents the capitate and the lunate from rubbing against each other. Final arthroscopic control ensures proper crossing of the tendon strands. RMC, radial midcarpal portal; UMC ulnar midcarpal portal.

The other end of the graft is then passed in an extra-articular way from the RMC portal to UMC portal, volar to the extensor tendons (Fig 3 and Fig 4). Lastly, using a mosquito, the PL is passed from RMC portal, crossing the first strand, to the rVC portal (Fig 5). Two separate capsular perforations must be made by the VC portal: one for the entry (uVC) and one for the exit of the tendon (rVC), so that the node remains pressed on the capsule. The graft is then knotted on itself, secured with a Vicryl 3-0 suture, and introduced in the midcarpal joint using a mosquito forceps. (Fig 6). An arthroscopic control is performed to ensure the correct position of the interposition (Fig 7).

### Postoperative Care

The wrist is immobilized immediately postoperatively. The postoperative plan would include strict immobilization for 30 days, then partial immobilization for 2 weeks with some wrist movement. At 45 days, the splint was permanently removed. Rehabilitation is initiated at approximately the sixth week.

## Discussion

At the radiocarpal level, tendon interpositions without bone excision allow a reduction of the conflict and improvement of functional signs.^7^ They play the role of a spacer and thus, strongly suppress articular friction. However, this technique is not definitive and does not remove mechanical forces as a CLA would, but it offers the advantage of relieving pain due to joint friction without stiffening the wrist. Therefore, it represents a means for rejecting more stiffening therapeutic indications, such as intracarpal arthrodesis.

Intracarpal arthrodesis are also difficult to perform, especially arthroscopically, and reoperation is not uncommon. A review of the literature, including 80 patients, revealed a reoperation rate of 14% and complication rate of 11%.^1^ For conflicts targeted on the capitolunate joint, CLA seems to be a more suitable indication than the four-corner arthrodesis because it is easier to perform, is less stiffening, and has a reduced need for bone grafts.^8^^,^^9^

Regarding the replacement of the proximal pole of the capitate, results are too uncertain and variable to confirm effectiveness,^4^^,^^5^ with the dislocation or poor position of the implant as a potential complication. However, this method is performed by open surgery, requiring a posterior arthrotomy and presenting a risk of stiffness. Subsequently, excision of the proximal pole of the capitate can be problematic during surgical revisions, requiring a significantly higher bone graft rate and difficult osteosynthesis with unsatisfactory results. Since the failure of CLA is high (nearly 20%),^10^ excision of the proximal pole could increase this risk.

One limitation of our study is the number of times this technique has been carried out, because of the limited number of cases. However, this technique offers a minimally invasive alternative using arthroscopy, and its conservative aspect allows a simplified return to traditional solutions that are stiffer, if the procedure fails. In addition, we performed our technique on rare pathologies to defer arthrodesis as far as possible. We believe that this technique could be applied to other capitolunate pathologies and, thus, offers alternatives to arthrodesis. Our technique presents the classic risks of wrist arthroscopies with the main drawback of performing a palmar approach, with the possible risks of nerve damage. In addition, if the posterior dissection is not performed correctly, it is possible that the interposing tendon will trap an extensor tendon, thus providing a tendon friction mechanism comparable to that of a finger pulley. Another criticism can be made if this technique is performed for cases of avascular necrosis of the head of the capitate, in that it does not treat the pathology but allows the surgeon more time to consider a less conservative surgery later and provides more stiffening. A surgeon must have good experience in wrist arthroscopy to perform this operation (Table 1).

**Table 1:** Surgical Pearls and Pitfalls

Pearls	Pitfalls
- When performing the palmar approach, it is necessary to remove all the tendons that are left on the radial side, except FDP 4 and 5, which are on the ulnar side. Be careful to leave fingers 4 and 5 without traction or place in a system that allows DIP flexion to recognize the correct tendons. - The tendon must pass from the palmar (VC portal) to the dorsal aspect by the UMC route, and then from the dorsal to the palmar aspect to ensure that the tendon node is palmar (can be troublesome at the dorsal aspect because it is close to the skin). - For the passage of the extensors, the first empty passage with mosquito forceps allows the creation of a space for dissection between the capsule and extensors, in order to avoid any risk of tendon caught in the interposition.	- To produce an optimal “hammock effect” under the head of the capitate, do not forget to make a crossed trajectory from palmar to dorsal aspect, and then from dorsal to palmar aspect

DIP, distal interphalangeal joint; FDP, flexor digitorum profundus; UMC midcarpal ulnar; VC, volar central.

## Supplementary Data

ICMJE author disclosure forms

Video 1 Arthroscopic midcarpal tendon interposition (AMTI) for avascular capitate necrosis. The patient is in supine position. The tourniquet is inflated to 250 mmHg. The traction is applied by a Finochietto hand or by Chinese fingers. Harvesting of the ipsilateral PL is performed using a stripper (Tendon Stripper, Arthrex). The graft is harvested through a small incision at the distal flexion crease of the wrist joint at the base of the carpal tunnel. A classic arthroscopic midcarpal exploration and debridement are performed by using RMC and UMC portals. Then, the palmar midcarpal portal is used to perform this interposition with two capsular perforations: one radial (rVC) and the other ulnar (uVC). The graft is introduced from the uVC portal to the RMC portal using mosquito forceps. The tendon can be introduced from uVC to RMC or in the opposite way for the first step without it being important for the remainder of the surgery. The graft is then passed in extra-articular way from the RMC portal to UMC portal. Then, the tendon is passed from UMC portal, crossing the first strand, to the rVC portal. The graft is then knotted on itself, secured with a suture, and introduced in the midcarpal joint using a mosquito forceps.
